# Epigenetic regulation in osteoarthritis: recent updates and emerging mechanisms

**DOI:** 10.3389/fgene.2026.1862466

**Published:** 2026-07-14

**Authors:** Yujin Zhao, Xianwen Liu

**Affiliations:** Department of Oral and Maxillofacial Surgery, Stomatological Hospital, School of Stomatology, Southern Medical University, Guangzhou, China

**Keywords:** DNA methylation, epigenetics, extracellular matrix, histone modification, multi-omics, non-coding RNA, osteoarthritis

## Abstract

Osteoarthritis (OA) is a common chronic degenerative joint disease characterized by progressive cartilage destruction, synovial inflammation, subchondral bone remodeling, and functional decline. Current treatments remain largely symptomatic and are unable to effectively halt or reverse disease progression. Increasing evidence indicates that epigenetic regulation provides a critical link between genetic susceptibility, mechanical loading, inflammation, aging, metabolic abnormalities, and pathological gene expression in OA. This review summarizes recent advances in OA epigenetics, with a particular focus on studies published over the past 2 years. We discuss classical mechanisms, including DNA methylation, histone modifications, and non-coding RNA-mediated regulation, and further highlight emerging epigenomic layers such as chromatin accessibility, enhancer and super-enhancer remodeling, three-dimensional genome organization, tissue-specific regulation, and the integration of genetics with single-cell and spatial multi-omics. These mechanisms contribute to inflammatory activation, chondrocyte metabolic imbalance, extracellular matrix degradation, programmed cell death, cellular senescence, oxidative stress, and abnormal inter-tissue crosstalk. Epigenetic biomarkers and epigenetic-based interventions, including extracellular vesicle-mediated delivery, engineered RNA therapeutics, and small-molecule epigenetic drugs, may offer new opportunities for early diagnosis, disease stratification, and precision therapy. However, current studies are limited by model heterogeneity, sample variability, insufficient causal validation, limited reproducibility, and translational challenges related to delivery and safety. Overall, epigenetic regulation provides a systematic framework for understanding OA heterogeneity and progression and may promote the development of disease-modifying therapeutic strategies.

## 1 Introduction

Osteoarthritis (OA) is one of the most common chronic degenerative joint diseases, clinically characterized by joint pain, restricted mobility, and functional decline, and it imposes a substantial burden on global public health systems (GBD 2021 Osteoarthritis Collaborators, 2023; Tang et al., 2025).

Despite the considerable disease burden of OA, current clinical treatments remain largely focused on pain relief, functional improvement, and symptomatic control, including nonsteroidal anti-inflammatory drugs, intra-articular injections, physical therapy, and joint replacement at the end stage (Tang et al., 2025). However, these approaches are still unable to fundamentally halt or reverse disease progression, and no disease-modifying OA drugs have yet been approved for routine clinical use (Jenei-Lanzl et al., 2025). Therefore, identifying molecular regulatory mechanisms that link environmental stimuli, cellular state transitions, and pathological phenotypes has become an important direction in OA research (Karsdal et al., 2025; Kim et al., 2025).

Epigenetic regulation provides a new conceptual framework for understanding the complex pathogenesis of OA (Ghezai et al., 2025). Epigenetics refers to molecular mechanisms that regulate chromatin states and gene expression programs without altering the underlying DNA sequence, mainly including DNA methylation, histone modifications, and non-coding RNA-mediated regulation (Barter et al., 2012; Ghezai et al., 2025). Compared with genetic mutations, epigenetic alterations are dynamic, reversible, and responsive to environmental cues, allowing them to reflect the long-term effects of mechanical stress, inflammatory stimulation, aging, and metabolic abnormalities on joint-resident cells (Barter et al., 2012; Nau et al., 2025). In OA, epigenetic dysregulation can influence chondrocyte phenotype maintenance, extracellular matrix metabolism (ECM), inflammatory responses, cell death, cellular senescence, subchondral bone remodeling, and synovial inflammation (Ghezai et al., 2025).

In recent years, OA epigenetic research has expanded from classical mechanisms to broader layers of epigenomic regulation (Bittner et al., 2024; Kramer et al., 2025). In addition to DNA methylation, histone acetylation/methylation, and non-coding RNAs(ncRNAs) such as miRNAs, lncRNAs, and circRNAs, increasing attention has been paid to chromatin accessibility, enhancer and super-enhancer activity, three-dimensional genome organization, and emerging histone acylation modifications. These emerging regulatory layers may help explain why certain OA-related genes establish stable pathological transcriptional programs after chronic inflammatory or mechanical stimulation (Bittner et al., 2024; Kramer et al., 2025).

Meanwhile, the development of single-cell epigenomics, spatial transcriptomics, spatial proteomics, spatial epigenomics, and multi-omics integration has provided new research paradigms for OA (Fan et al., 2024a; Fan et al., 2024b; Liu et al., 2025d). Traditional bulk tissue-based studies often reflect average molecular changes and are limited in distinguishing regulatory features across different tissues, cell subpopulations, and spatial regions. Technologies such as single-cell RNA sequencing and single-cellassay for transposase-accessible chromatin with sequencing (ATAC-seq) can reveal the transcriptional and chromatin regulatory states of distinct cell populations, including homeostatic chondrocytes, inflammatory chondrocytes, hypertrophic-like chondrocytes, senescent-like chondrocytes, synovial fibroblasts, macrophages, and subchondral bone-associated cells (Fan et al., 2024b; Liu et al., 2025c; Raut et al., 2025b). Spatial omics technologies further enable the analysis of local regulatory networks in cartilage zones, synovial inflammatory regions, cartilage lesion margins, and subchondral bone remodeling areas while preserving tissue architecture (Fan et al., 2024a; Fan et al., 2024b). These advances are shifting OA research from single-gene or single-pathway analysis toward cell type-specific, spatially resolved, and multilayered regulatory network analysis (Fan et al., 2024b; Liu et al., 2025d).

Based on this background, this review summarizes recent advances in epigenetic regulation in OA, with a focus on the roles of DNA methylation, histone modifications, and ncRNAs in disease initiation and progression. In addition, we further discuss emerging directions, including chromatin accessibility, enhancers and super-enhancers, three-dimensional genome organization, tissue-specific regulation, and integration of genetics with single-cell and spatial multi-omics. Rather than simply listing individual molecules, this review emphasizes how epigenetic alterations regulate gene expression networks, cellular state transitions, inter-tissue crosstalk, and disease heterogeneity in OA. Furthermore, we discuss current limitations related to model heterogeneity, reproducibility, causal validation, and clinical translation, aiming to provide a reference for mechanistic refinement and precision medicine applications in OA epigenetic research.

## 2 Pathological context of OA relevant to epigenetic regulation

OA is a chronic degenerative disease that affects the whole joint organ (Tang et al., 2025). Its initiation and progression are not driven by a single pathological event, but rather by the combined effects of abnormal mechanical loading, inflammatory stimulation, cellular senescence, metabolic imbalance, and aberrant crosstalk among joint tissues (Xia et al., 2024a; Tang et al., 2025). Traditionally, OA has been regarded primarily as a disease characterized by cartilage wear and extracellular matrix degradation. However, accumulating evidence indicates that OA should be understood as a complex pathological condition involving multiple joint tissues, including articular cartilage, subchondral bone, synovium, meniscus, and infrapatellar fat pad (Li et al., 2024a; Tang et al., 2025). During this process, environmental cues and intracellular signaling alterations continuously act on joint-resident cells and reshape gene expression programs through epigenetic mechanisms, thereby influencing disease initiation, progression, and heterogeneity (Caldo et al., 2024; Hatzikotoulas et al., 2025). Therefore, reconsidering OA pathogenesis from an epigenetic perspective may help clarify the molecular links among environmental stimuli, cellular states, and disease phenotypes (Ghezai et al., 2025; Hatzikotoulas et al., 2025).

Articular cartilage degeneration is one of the most representative pathological features of OA, and it essentially reflects an imbalance between anabolic and catabolic activities in chondrocytes (Goldring and Marcu, 2012). Under physiological conditions, chondrocytes maintain cartilage mechanical strength and elasticity by preserving the expression of ECM components, such as type II collagen and aggrecan (Goldring and Marcu, 2012). However, under stimulation by inflammatory cytokines, abnormal mechanical stress, and oxidative stress, chondrocytes gradually acquire an abnormal phenotype characterized by reduced matrix synthesis, increased expression of matrix-degrading enzymes, and enhanced hypertrophic-like differentiation (Bloks et al., 2024). Importantly, the aberrant activation of these transcriptional programs is not merely the consequence of transient signaling pathway activation, but may also be closely associated with DNA methylation, histone modifications, ncRNA regulation, and alterations in chromatin accessibility (Caldo et al., 2024; Roberts and Rice, 2024; Kramer et al., 2025). For example, genomic regions associated with cartilage matrix synthesis and degradation may undergo promoter methylation changes, enhancer remodeling, or abnormal deposition of repressive histone marks, thereby maintaining chondrocytes in a long-lasting catabolic, inflammatory, or degenerative state (Liu et al., 2024b; Roberts and Rice, 2024; Kramer et al., 2025). Thus, cartilage degeneration is not only a structural manifestation of OA, but also an important entry point for understanding how epigenetic regulation reshapes cell fate and gene expression homeostasis (Kramer et al., 2025).

Inflammation represents a critical link between environmental stimuli and epigenetic reprogramming. Although OA is not a typical autoimmune inflammatory disease, low-grade, chronic, and persistent inflammation plays an essential role in disease progression. Pro-inflammatory cytokines, including interleukin-1β (IL-1β), tumor necrosis factor-α (TNF-α), and interleukin-6 (IL-6), can activate signaling pathways such as nuclear factor kappa-B (NF-κB), mitogen-activated protein kinase (MAPK), and Janus kinase/signal transducer and activator of transcription (JAK/STAT), leading to the induction of inflammatory mediators, chemokines, and matrix-degrading enzymes (Molnar et al., 2021; Fazio et al., 2024). More importantly, persistent inflammatory stimulation may further alter chromatin states, rendering promoter or enhancer regions near inflammation-related genes more accessible and transcriptionally active, thereby establishing a sustained pathological transcriptional program (Caldwell and Li, 2024; Roberts and Rice, 2024). For instance, transcription factors such as NF-κB and activator protein-1 (AP-1) not only directly regulate inflammatory gene expression, but may also interact with histone acetyltransferases (HATs), methyltransferases, or chromatin remodeling complexes to influence the distribution of epigenetic marks such as H3K27ac, H3K4me1, and H3K9me3 (Caldwell and Li, 2024). These findings suggest that inflammation in OA is not simply a transient activation of signaling pathways, but may also involve the formation of more stable epigenetic memory (Caldwell and Li, 2024).

Cellular senescence also provides an important pathological context for epigenetic regulation in OA (Diekman and Loeser, 2024; Han et al., 2024). With aging or prolonged exposure to inflammation, mechanical injury, and oxidative stress, chondrocytes and other joint-resident cells may gradually enter a senescent state, characterized by reduced proliferative capacity, altered stress responses, and enhanced senescence-associated secretory phenotype (SASP) (Diekman and Loeser, 2024; Han et al., 2024). Senescent cells secrete inflammatory cytokines, chemokines, and matrix-degrading enzymes, thereby aggravating local inflammation and ECM destruction and forming a vicious cycle that promotes OA progression (Han et al., 2024). From an epigenetic perspective, cellular senescence is frequently accompanied by alterations in heterochromatin organization, DNA methylation drift, abnormal changes in repressive histone marks such as H3K9me3 and H3K27me3, and remodeling of ncRNA expression profiles (Zhang et al., 2021; Crouch et al., 2022; Dasgupta et al., 2024). These changes may lead to aberrant activation of previously silenced inflammatory, stress-responsive, and catabolic genes, while suppressing protective genes required for the maintenance of chondrocyte homeostasis (Zhang et al., 2021; Diekman and Loeser, 2024). Therefore, senescence is not only an important risk factor for OA, but also a key epigenetic background for understanding chronic disease progression and pathological memory (Dasgupta et al., 2024; Diekman and Loeser, 2024; Han et al., 2024).

Beyond cartilage, subchondral bone and synovium also contribute to OA development and progression and may exhibit distinct epigenetic regulatory features (Hatzikotoulas et al., 2025; Tang et al., 2025). Subchondral bone remodeling can alter the local biomechanical environment of the joint, thereby affecting chondrocyte metabolism and cartilage integrity (Hu et al., 2021; Chen et al., 2024b). Synovial inflammation can influence cartilage and subchondral bone through the secretion of inflammatory cytokines, chemokines, and extracellular vesicles (Tang et al., 2024). Because different joint tissues contain distinct cell populations and are exposed to different mechanical and inflammatory microenvironments, their epigenetic alterations are likely to be tissue-specific (Zhang et al., 2016; Hatzikotoulas et al., 2025). For example, epigenetic changes in cartilage may be more directly related to ECM homeostasis and the maintenance of chondrocyte phenotype, whereas epigenetic regulation in subchondral bone may involve osteogenesis, osteoclastogenesis, angiogenesis, and osteochondral crosstalk (Zhang et al., 2016; Hu et al., 2021). In synovial tissues, epigenetic dysregulation may be more closely associated with inflammatory amplification and immune microenvironment remodeling (Tang et al., 2024; Hatzikotoulas et al., 2025). Therefore, viewing OA as a whole-joint disease and comparing epigenetic features across different tissue types are essential for understanding disease heterogeneity and identifying tissue-specific therapeutic targets (Hatzikotoulas et al., 2025; Tang et al., 2025).

It should be noted that OA pathology is characterized by marked tissue and cellular heterogeneity (Raut et al., 2025b). Although traditional bulk tissue-based studies can reveal average molecular alterations, they are limited in distinguishing regulatory features across different joint tissues, cell subpopulations, and spatial regions (Fan et al., 2024a; Fan et al., 2024b). For example, chondrocytes in the superficial, middle, and deep zones of cartilage differ in mechanical loading, metabolic status, and inflammatory responses, while cells in the synovium, subchondral bone, and adipose tissues may exhibit distinct epigenetic states (Tang et al., 2024; Raut et al., 2025b). Emerging technologies such as single-cell epigenomics, single-cell ATAC-seq, spatial transcriptomics, and spatial epigenomics therefore provide important tools for dissecting cell type-specific and spatially resolved epigenetic regulatory networks in OA (Fan et al., 2024a; Kramer et al., 2025). These approaches may shift OA research from average changes at the bulk tissue level toward precise analysis at the single-cell and spatial microenvironment levels (Fan et al., 2024a; Fan et al., 2024b; Kramer et al., 2025).

Overall, OA-related pathological processes provide a complex stimulus background for the initiation and maintenance of epigenetic alterations. Mechanical loading, inflammatory cytokines, aging, oxidative stress, and metabolic abnormalities may collectively act on joint-resident cells and reshape gene expression networks through multilayered mechanisms, including DNA methylation, histone modifications, ncRNAs, chromatin accessibility, and three-dimensional genome organization. Compared with traditional studies focusing on individual signaling pathways, epigenetic regulation may better explain the persistence, tissue specificity, and inter-individual variability of pathological states in OA. Based on this framework, the following sections will further discuss the roles of DNA methylation, histone modifications, ncRNAs, and emerging epigenomic mechanisms in OA pathogenesis, disease stratification, and potential therapeutic development.

## 3 Epigenetic in OA

**FIGURE 1:**
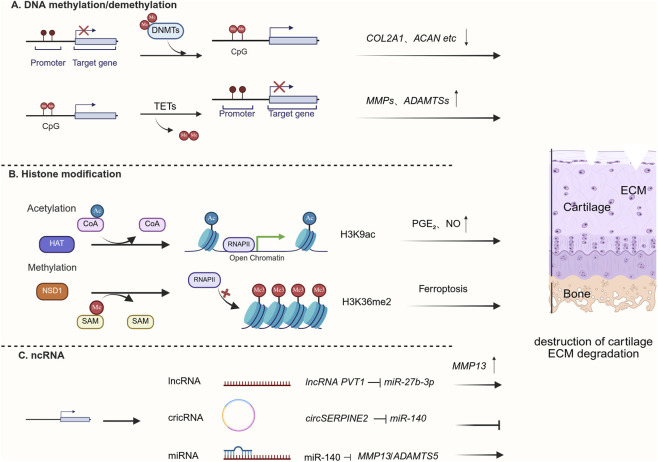
Epigenetic regulation in OA pathogenesis. Epigenetic mechanisms, including DNA methylation, histone modifications, and ncRNAs, regulate gene transcription and contribute to OA progression **(A)** DNA methylation/demethylation. DNMTs mediate CpG methylation and repress the transcription of cartilage matrix-related genes, such as *COL2A1* and *ACAN*, whereas TET enzymes promote DNA demethylation and activate the transcription of matrix-degrading enzymes, such as *MMPs* and *ADAMTSs*, thereby contributing to ECM degradation **(B)** Histone modifications. Histone acetylation mediated by HATs induces chromatin relaxation and enhances transcriptional activation through RNAPII. For example, H3K9ac is associated with increased expression of inflammatory mediators, including PGE_2_ and NO. Histone methylation, such as H3K36me2, also regulates gene expression and may be involved in chondrocyte ferroptosis **(C)** ncRNAs. lncRNAs, circRNAs, and miRNAs form regulatory networks that modulate cartilage homeostasis and ECM metabolism. For instance, *lncRNA PVT1* regulates *MMP13* by sponging *miR-27b-3p*, whereas *circSERPINE2* interacts with *miR-140* to influence ECM metabolism. *miR-140* directly targets *MMP13* and *ADAMTS5*. Overall, these epigenetic mechanisms promote inflammation, cartilage degeneration, and ECM breakdown, thereby contributing to OA progression. Created in BioRender. jin, Y. (2026) https://BioRender.com/qgya2jp.

Recent studies have demonstrated that epigenetic regulation plays an important role in the initiation and progression of OA (Shen et al., 2017; Rice et al., 2020; Núñez-Carro et al., 2023). Unlike genetic mutations, epigenetic mechanisms regulate gene expression without altering the DNA sequence, thereby influencing chondrocyte function and joint tissue homeostasis (Jeffries et al., 2014; Rice et al., 2020). Factors such as long-term mechanical stress, inflammatory stimulation, and aging can alter the epigenetic status of chondrocytes, subsequently leading to metabolic imbalance in cartilage and inflammatory responses (Shen et al., 2017; Young et al., 2022). Currently, OA-related epigenetic regulation is generally considered to include DNA methylation, histone modifications, and non-coding RNA-mediated regulation (Núñez-Carro et al., 2023). These mechanisms collectively participate in the development and progression of OA by regulating inflammatory signaling pathways and extracellular matrix (ECM) metabolism, and they may form complex regulatory networks (Shen et al., 2017). Therefore, in-depth investigation of epigenetic mechanisms may help elucidate the molecular basis of OA and identify novel potential therapeutic targets (Núñez-Carro et al., 2023; Ghezai et al., 2025) (Figure 1).

### 3.1 DNA methylation in OA

DNA methylation is an important epigenetic modification that regulates gene transcriptional activity by adding a methyl group to cytosine residues to form 5-methylcytosine (5-mC) (Jones, 2012; Moore et al., 2013). This process is primarily catalyzed by DNA methyltransferases (DNMTs) (DNMT1, DNMT3A, and DNMT3B), among which DNMT3A and DNMT3B are responsible for establishing new methylation patterns, while DNMT1 maintains existing methylation states (Okano et al., 1999; Moore et al., 2013). In addition, enzymes of the ten-eleven translocation (TET) family can oxidize 5-mC to 5-hydroxymethylcytosine (5-hmC), thereby participating in the process of DNA demethylation (Tahiliani et al., 2009). DNA methylation typically occurs at cytosine-phosphate-guanine (CpG) sites, particularly within CpG islands located in promoter regions, where the methylation status can regulate gene expression by influencing the binding of transcription factors (Jones, 2012; Miranda-Duarte, 2018).

Recent studies have demonstrated that aberrant DNA methylation plays an important role in the initiation and progression of OA. Multiple studies have found that the expression of DNA methylation enzymes and overall methylation levels are significantly altered in OA cartilage and are closely associated with cartilage degeneration and inflammatory responses. For example, hypermethylation of the glutathione peroxidase 4 (*GPX4*) promoter region can suppress its expression, thereby aggravating oxidative stress and ferroptosis and promoting OA progression (Feng et al., 2024). Meanwhile, increased expression of DNMT1 and DNMT3A in hip OA cartilage suggests a key role of DNA methylation in disease progression (Kawarai et al., 2024). In addition, as OA severity increases, the number of differentially methylated regions in cartilage tissues significantly rises and is closely associated with abnormal expression of cartilage degeneration-related genes (Kreitmaier et al., 2024)Some studies have also proposed that site-specific methylation has potential diagnostic value; for instance, methylation of CpG sites associated with bone morphogenetic protein 7 (*BMP7*) may serve as a biomarker for hand OA (Kuroiwa et al., 2025).

DNA demethylation also participates in the regulation of OA-related genes. Studies have shown that demethylation of promoter regions in cartilage degradation-related genes can promote transcription factor binding and activate gene expression. For example, demethylation of the *ADAMTS-5* promoter enhances SPI-1 binding and accelerates cartilage matrix degradation (He et al., 2024), Similarly, demethylation of the *MMP-3* promoter activates its expression by facilitating STAT4 binding and thereby exacerbates cartilage degeneration (Kong et al., 2025). Furthermore, synergistic interactions exist among different epigenetic modifications; for example, DNA demethylation and changes in histone H3K9 methylation jointly regulate *ADAMTS-5* expression (Chen et al., 2024d).

In addition to regulating gene expression, DNA methylation is closely associated with genetic susceptibility and environmental factors. For instance, promoter methylation can regulate the expression of OA susceptibility genes and influence disease risk (Roberts et al., 2024). Factors such as mechanical stress, metabolic abnormalities, and environmental exposures during embryonic development may also influence OA development by altering DNA methylation patterns (Bloks et al., 2024). Moreover, methylation profiles established during articular cartilage development are believed to potentially determine susceptibility to OA in adulthood (McDonnell et al., 2024).

Overall, DNA methylation participates in the pathological processes of OA by regulating multiple mechanisms, including inflammatory responses, oxidative stress, and chondrocyte metabolism. With the continuous advancement of epigenomic research, DNA methylation not only provides important insights into the molecular mechanisms of OA but also offers potential directions for the development of novel diagnostic biomarkers and targeted therapeutic strategies.

### 3.2 Histone modifications in OA

Histone modifications are important epigenetic regulatory mechanisms that regulate gene expression by altering chromatin structure and transcriptional activity, and they play a key role in the development and progression of OA. Recent studies have shown that histone modifications participate in chondrocyte dysfunction and alterations of the joint tissue microenvironment by regulating processes such as inflammatory responses, cell death, energy metabolism, and extracellular matrix (ECM) homeostasis. Among these mechanisms, the dynamic balance between histone acetylation and deacetylation is considered an important component of epigenetic regulation in OA. For example, ECM stiffening can suppress the expression of histone deacetylase 3 (HDAC3), leading to abnormal histone hyperacetylation and promoting chondrocyte senescence, thereby accelerating OA progression (Fu et al., 2024a). In addition, members of the histone deacetylase (HDAC) family exhibit significant functional heterogeneity in OA; among them, histone deacetylase 4 (HDAC4) has been demonstrated to exert important chondroprotective effects by suppressing chondrocyte apoptosis through regulation of the activating transcription factor 4-dependent endoplasmic reticulum stress pathway, thereby alleviating OA phenotypes (Gu et al., 2024). In contrast, histone deacetylase 6 (HDAC6) contributes to OA progression by promoting inflammatory signaling activation and ECM degradation, and its specific inhibitor Tubastatin A has been shown to significantly alleviate related pathological changes (Zhou et al., 2025). Furthermore, the sirtuin family deacetylase sirtuin 1 (Sirt1) also plays a protective role in maintaining chondrocyte homeostasis by regulating ferroptosis and mitochondrial function, thereby delaying OA progression (Xiong et al., 2025).

In addition to acetylation, histone methylation also plays an important role in the pathological regulation of OA. Studies have shown that methyltransferases and demethylases influence inflammatory responses, metabolic reprogramming, and programmed cell death in chondrocytes by regulating key epigenetic marks such as histone H3 lysine 27 trimethylation (H3K27me3) and histone H3 lysine nine trimethylation (H3K9me3). For example, aberrant histone H3 lysine 36 dimethylation (H3K36me2) mediated by the methyltransferase nuclear receptor binding SET domain protein 1 (NSD1) can induce ferroptosis in chondrocytes and accelerate OA progression (Guan et al., 2024). SET domain containing lysine methyltransferase 7 (SETD7) participates in OA development by regulating inflammatory responses and autophagy processes (Yuan et al., 2025). Meanwhile, histone demethylases also exhibit distinct regulatory roles in OA; for instance, lysine demethylase 6 B (KDM6B) is considered to have protective effects, whereas lysine demethylase 3A (KDM3A) may promote OA progression (Yang et al., 2024a; Fu et al., 2025). In addition, dynamic alterations in key methylation marks are closely associated with OA, and abnormal H3K27me3 and H3K9me3 have been considered important factors driving the expression of inflammation- and senescence-related genes (Wang et al., 2025b; Zheng et al., 2025).

Beyond classical histone acetylation and methylation, emerging histone acylation modifications, such as lactylation and malonylation, provide new perspectives for understanding the link between metabolic reprogramming and epigenetic regulation in OA (Dai et al., 2020; Xu et al., 2024). Histone malonylation is closely associated with malonyl-CoA, mitochondrial metabolism, and the tricarboxylic acid cycle (Nishida et al., 2015; Zhang et al., 2023). Members of the sirtuin family, especially sirtuin 5 (Sirt5), possess demalonylase activity and may influence cellular function by regulating mitochondrial metabolism and chromatin states (Nishida et al., 2015; Zhang et al., 2023). Although direct evidence supporting the involvement of malonylation in OA remains limited, the mitochondrial dysfunction, oxidative stress, and metabolic abnormalities commonly observed in OA suggest that malonylation may represent a new direction linking metabolic disturbance with chromatin regulation (Dai et al., 2020; Liu et al., 2025). Overall, lactylation and malonylation indicate that metabolic abnormalities in OA may reshape pathological transcriptional programs by altering histone modification patterns (Dai et al., 2020; Xu et al., 2024). However, direct evidence remains insufficient, and future studies should integrate metabolomics, histone modification profiling, chromatin immunoprecipitation sequencing (ChIP-seq), cleavage under targets and tagmentation (CUT&Tag), and transcriptomics to further validate their specific roles and causal significance (Liu et al., 2025; Xu et al., 2024).

Histone lactylation is mainly associated with enhanced glycolysis and lactate accumulation (Xia et al., 2024a; Xu et al., 2024). Lactate dehydrogenase A (LDHA) can influence lactylation levels by regulating lactate production (Xia et al., 2024a). Recent studies have further identified specific histone lactylation marks, such as histone H3 lysine 18 lactylation (H3K18la) and H4K12la, as representative modifications linking lactate metabolism to transcriptional regulation (Pan et al., 2022; Xia et al., 2024a). In OA, LDHA-mediated H3K18la has been reported to enhance the transcriptional activity of glycolysis-related genes, suggesting that lactylation may participate in a metabolic–epigenetic feedback loop during cartilage degeneration (Pan et al., 2022; Xia et al., 2024a). In addition, histone lactylation is dynamically regulated by lactylation “writers” and “erasers”; p300/CBP has been proposed as an important lactyltransferase (Zhang et al., 2019; Dong et al., 2024), whereas class I histone deacetylases, particularly HDAC1–3, and some sirtuin family members may function as delactylases in a context-dependent manner (Moreno-Yruela et al., 2022; Zu et al., 2022). In the OA microenvironment, inflammatory stimulation, hypoxia-like stress, mitochondrial dysfunction, and abnormal mechanical loading may promote lactate accumulation and regulate the expression of genes related to inflammatory responses, matrix catabolism, cellular senescence, and ferroptosis through lactylation (Xia et al., 2024a; Zhang et al., 2025). Notably, emerging evidence suggests that lactylation may also be linked to chondrocyte ferroptosis by regulating ferroptosis-related transcriptional programs, lipid peroxidation, reactive oxygen species accumulation, and iron metabolism imbalance (Xia et al., 2024a; Zhang et al., 2025). For example, lactate dehydrogenase-mediated histone lactylation has been associated with ferroptosis-related changes in OA chondrocytes, indicating that the lactate–lactylation axis may connect metabolic reprogramming with oxidative damage and cartilage degeneration (Xia et al., 2024a; Zhang et al., 2025). However, the role of lactylation is not unidirectionally pro-inflammatory or purely pathogenic (Xu et al., 2024). Depending on cell type, disease stage, and metabolic microenvironment, lactylation may also participate in metabolic adaptation, immune regulation, and tissue repair (Dai et al., 2020; Xu et al., 2024). Therefore, histone lactylation should be interpreted as a context-dependent epigenetic modification rather than a simple pro-inflammatory signal. Excessive or persistent lactylation may amplify inflammatory, catabolic, senescence-related, and ferroptosis-related responses, whereas balanced or adaptive lactylation may contribute to metabolic adaptation, immune modulation, and tissue repair.

**FIGURE 2:**
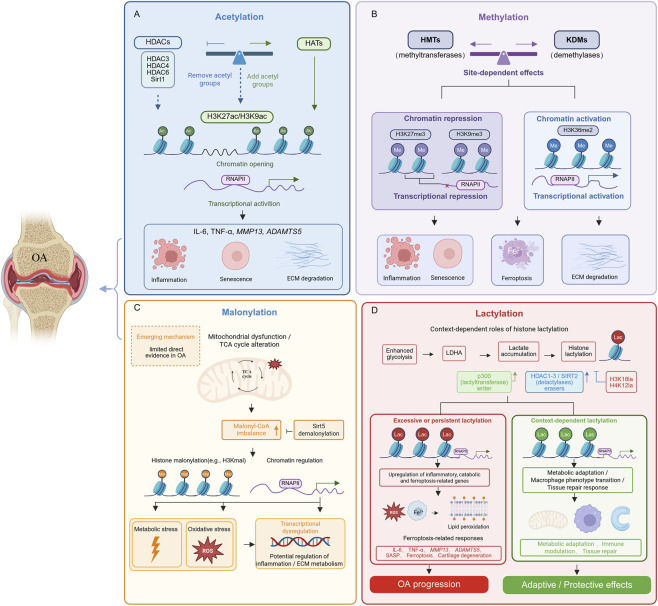
Classical and emerging histone modifications in OA. Histone modifications regulate chromatin structure and transcriptional activity and contribute to OA progression by modulating inflammation, chondrocyte senescence, cell death, and ECM metabolism **(A)** Histone acetylation. Histone acetylation generally promotes chromatin opening and transcriptional activation. Dysregulated HAT/HDAC balance may regulate inflammatory activation, cellular senescence, and ECM degradation in OA **(B)** Histone methylation. Histone methylation can either activate or repress transcription depending on the modified residue and methylation state. Altered histone marks, such as H3K27me3, H3K9me3, and H3K36me2, may influence inflammation, senescence, ferroptosis, and cartilage matrix metabolism **(C)** Histone malonylation. Histone malonylation may link mitochondrial dysfunction, TCA cycle alterations, and malonyl-CoA metabolism to chromatin regulation. However, direct evidence for its role in OA remains limited and requires further validation **(D)** Histone lactylation. Histone lactylation connects glycolytic reprogramming and lactate accumulation with transcriptional regulation. Representative lactylation marks, such as H3K18la and H4K12la, may link lactate metabolism to the activation of OA-related transcriptional programs. Histone lactylation is dynamically regulated by lactylation writers and erasers, including p300/CBP as potential lactyltransferases and HDAC1–3 or SIRT2 as context-dependent delactylases. Excessive or persistent lactylation may promote inflammatory, catabolic, senescence-associated, or ferroptosis-related programs, partly through oxidative stress, iron accumulation, lipid peroxidation, and ferroptosis-related gene regulation, whereas context-dependent lactylation may also participate in metabolic adaptation, immune modulation, macrophage phenotype transition, and tissue repair. Overall, classical and emerging histone modifications form an important epigenetic regulatory layer linking metabolic disturbance, inflammatory activation, and cartilage degeneration in OA. Created in BioRender. jin, Y. (2026) https://BioRender.com/bwk9qtd.

Overall, histone modifications regulate inflammatory responses, cell death, and metabolic homeostasis at multiple levels, constituting an important component of the epigenetic regulatory network in OA (Figure 2). A deeper understanding of the specific roles of different histone modifications and their associated enzymes across various cell types and disease stages will not only help elucidate the molecular mechanisms of OA but also provide an important theoretical basis for developing novel therapeutic strategies targeting epigenetic regulation.

### 3.3 Non-coding RNAs in OA

Non-coding RNAs (ncRNAs) are a class of RNA molecules that do not encode proteins but are capable of regulating gene expression, among which microRNAs (miRNAs) and long non-coding RNAs (lncRNAs) are the most representative (Bartel, 2009; Esteller, 2011). miRNAs suppress translation or promote degradation of target mRNAs through complementary binding, whereas lncRNAs regulate gene expression at both transcriptional and post-transcriptional levels by interacting with chromatin-modifying complexes, transcription factors, or miRNAs (Bartel, 2009; Rinn and Chang, 2012). Recent studies have demonstrated that ncRNAs play important regulatory roles in the initiation and progression of OA, particularly in key biological processes such as inflammatory responses in chondrocytes, cell death, extracellular matrix (ECM) degradation, and metabolic homeostasis (Kung et al., 2013).

#### 3.3.1 IncRNA

In recent years, numerous studies have revealed important regulatory roles of lncRNAs in OA. lncRNAs can regulate the miRNA-mRNA axis through competitive endogenous RNA (ceRNA) networks, thereby influencing inflammatory responses and cartilage metabolism. For example, *CRNDE* suppresses inflammatory cytokine release and reduces matrix degradation through the *miR-31*/NF-κB axis (Liu et al., 2024a), and *NKILA* can directly inhibit the NF-κB signaling pathway to alleviate inflammatory responses (Wang et al., 2024a). In addition, some lncRNAs participate in OA progression by regulating cellular metabolism and mitochondrial function. For instance, *OIP5-AS1* enhances mitophagy and improves chondrocyte injury through the AMP-activated protein kinase/protein kinase B/mammalian target of rapamycin (AMPK/Akt/mTOR) signaling pathway (Sun et al., 2024b). In terms of cell death regulation, *EMBP1* promotes NLRP3-mediated pyroptosis by regulating the *miR-454-3p*/interferon regulatory factor 1 (IRF1) axis through a ceRNA mechanism (Ma et al., 2025). These findings suggest an important role of lncRNAs in inflammatory cell death.

lncRNAs also play key roles in cartilage metabolism and immune-inflammatory regulation. Some lncRNAs maintain cartilage homeostasis by regulating signaling pathways such as phosphoinositide 3-kinase/protein kinase B (PI3K/Akt). For example, *SNHG1* suppresses excessive autophagy and alleviates cartilage injury by activating the PI3K/Akt signaling pathway (Xu et al., 2024), whereas *SNHG7* can inhibit ferroptosis and inflammatory responses through exosomal delivery regulating the *miR-485-5p*/ferroptosis suppressor protein 1 (FSP1) axis [48]. In addition, lncRNAs such as *MEG3* and *HOXA11-AS* have also been shown to participate in OA regulation by modulating oxidative stress or ferroptosis-related pathways (Zhang et al., 2024c; Zhu et al., 2024a).

Notably, not all lncRNAs exert protective effects; some instead promote OA progression. For example, *FAS-AS1* enhances transcript stability through METTL14-mediated methylation modification and promotes inflammatory responses (Zhang et al., 2024b), whereas *H19* promotes abnormal subchondral bone remodeling by activating the PI3K/protein kinase B/glycogen synthase kinase 3 (PI3K/AKT/GSK3) signaling pathway (Han et al., 2024). In addition, lncRNAs such as *LINC00958* and *LINC00511* promote inflammatory responses and pyroptosis in chondrocytes by regulating inflammatory signaling pathways or the NLRP3 inflammasome (Yin et al., 2024; Zhai et al., 2024).

Beyond their involvement in OA pathogenesis, lncRNAs also exhibit potential applications in disease diagnosis and therapy. For example, the expression levels of *ITGB2-AS1* and intercellular adhesion molecule 1 (ICAM-1) in the serum of OA patients are significantly associated with disease severity. This suggests their potential value as biomarkers (Selim et al., 2024). Furthermore, some lncRNAs are involved in pain regulation and autophagy modulation. For instance, *XIST* alleviates OA-related pain by regulating the expression of Nav1.7 (Fu et al., 2024b), while *SIRT1-AS* and *RP13-516M14.1* exert protective effects by regulating cartilage-degrading enzyme expression or autophagy processes, respectively (Tokura et al., 2025; Yang et al., 2026). Meanwhile, other ncRNAs such as circular RNAs(circRNAs) also contribute to the formation of complex regulatory networks. For example, *circPVT1* regulates the *miR-550a-3p*/C-X3-C motif chemokine receptor 1 (CX3CR1) axis through a ceRNA mechanism, thereby influencing chondrocyte inflammation and matrix metabolism (Liu et al., 2025b).

Overall, lncRNAs participate in the development and progression of OA through multiple mechanisms, including ceRNA networks, signaling pathway regulation, and epigenetic modulation. Their bidirectional roles in regulating inflammation, cell death, and metabolic homeostasis highlight the complexity of ncRNA regulatory networks. Mechanistically, lncRNAs should not be viewed merely as isolated upstream or downstream regulators of individual molecules; rather, they integrate OA-related pathological signals through multilayered regulatory modes. First, lncRNAs can function as ceRNA sponges that sequester specific miRNAs, thereby relieving miRNA-mediated repression of target mRNAs and influencing inflammatory cytokine release, the expression of extracellular matrix-degrading enzymes, and chondrocyte fate. Second, some lncRNAs may regulate target gene transcriptional activity or transcript stability by recruiting chromatin-modifying complexes or influencing RNA methylation-related processes, thus contributing to epigenetic reprogramming in OA. In addition, lncRNAs can transmit regulatory information among chondrocytes, synovial cells, subchondral bone cells, and immune cells through exosomes, thereby participating in cross-tissue and cross-cellular inflammatory and metabolic regulatory networks. More importantly, lncRNAs form feedback regulatory relationships with NF-κB, PI3K/Akt, AMPK/mTOR, NLRP3 inflammasome, and ferroptosis-related pathways, making them important molecular nodes that connect inflammatory stimulation, cell death, mitochondrial function, and cartilage matrix homeostasis. With the continued advancement of transcriptomic and functional studies, lncRNAs not only provide new perspectives for understanding the molecular mechanisms of OA but also offer significant potential for the development of novel diagnostic biomarkers and targeted therapeutic strategies.

#### 3.3.2 circRNA

In recent years, circular RNAs (circRNAs), a class of ncRNAs characterized by a covalently closed-loop structure, have attracted considerable attention in OA due to their high stability, evolutionary conservation, and diverse regulatory mechanisms (Kristensen et al., 2019; Zhou et al., 2019). Studies have shown that circRNAs mainly function as competitive endogenous RNAs (ceRNAs), regulate signaling pathways, and influence protein interactions, thereby participating in key pathological processes such as inflammatory responses, chondrocyte apoptosis, and extracellular matrix (ECM) metabolism, ultimately playing important regulatory roles in OA development and progression (Li et al., 2015; Meng et al., 2017; Zhou et al., 2019).

In the regulation of inflammation, circRNAs exhibit notable bidirectional regulatory effects. For example, *circUbqln1* can interact with the 14-3-3ζ protein to upregulate proline dehydrogenase (PRODH) expression, thereby aggravating inflammatory responses and promoting cartilage degeneration (Feng et al., 2024); *circMYO1C* enhances inflammatory responses and induces chondrocyte injury through the N6-methyladenosine (m6A)/HMGB1 axis (Sun et al., 2024a). In contrast, some circRNAs exhibit anti-inflammatory and protective effects. For instance, *circYAP1* derived from bone marrow mesenchymal stem cell (BMSC) exosomes can alleviate inflammatory injury by regulating the *miR-21*/Toll-like receptor 7 (TLR7) signaling pathway (El-Din et al., 2024). Additionally, *circPAFAH1B2* and *circ_0075048* have also been shown to participate in the regulation of the inflammatory microenvironment and cartilage injury in OA (Bu et al., 2025; Cai et al., 2025).

In the regulation of chondrocyte apoptosis and survival, different circRNAs also exhibit significant functional differences. For example, *circSEC24A* promotes chondrocyte apoptosis and accelerates OA progression through the *miR-107-5p*/caspase 3 (CASP3) or *miR-26b-5p*-related pathways (Dadihanc et al., 2024; Wang et al., 2024c). Conversely, *circ_0044235* suppresses chondrocyte apoptosis and inflammatory responses through the *miR-375*/phosphoinositide-3-kinase regulatory subunit 3 (PIK3R3) axis (Qian et al., 2024a) while *circTRIM25* promotes chondrogenesis through the *miR-138-5p*/cAMP response element-binding protein 1 (CREB1) axis, demonstrating potential therapeutic value (He et al., 2024). In addition, *circPVT1* is highly expressed in OA cartilage and regulates cell proliferation and inflammatory responses through the *miR-550a-3p*/CX3CR1 axis, which is associated with poor disease prognosis (Liu et al., 2025b).

circRNAs also contribute to cartilage homeostasis by regulating ECM metabolism. For example, *circPDE1C* and *hsa_circ_0045474* promote ECM degradation through the *miR-766-3p*/SGTB axis and the *miR-485-3p*/transcription factor 4 (TCF4) pathway, respectively (Gao et al., 2024; You et al., 2024). In contrast, *circSLTM* and *circACAP2* inhibit matrix degradation and improve chondrocyte function by regulating the *miR-515-5p*/VAPB axis or the *miR-21-5p*/pleomorphic adenoma gene 1 (PLAG1) axiss (Chen et al., 2024a; Chen et al., 2024c).

Overall, circRNAs regulate inflammatory responses, cell apoptosis, and ECM metabolism through ceRNA networks and multiple signaling pathways, playing important roles in the ncRNA regulatory network of OA. They may exert both pathogenic and protective effects depending on the specific regulatory context. Mechanistically, the role of circRNAs in OA is not limited to acting as miRNA sponges that regulate individual miRNA-mRNA axes; rather, circRNAs participate in the broader remodeling of cartilage and the joint microenvironment through multiple regulatory modes. First, circRNAs can sequester miRNAs through ceRNA mechanisms, thereby indirectly regulating the expression of inflammatory cytokines, ECM-degrading enzymes, apoptosis-related proteins, and chondrogenesis-associated transcription factors, ultimately influencing chondrocyte inflammatory responses, matrix metabolism, and cell fate. Second, some circRNAs can interact with RNA-binding proteins or signaling-related proteins, affecting protein stability, subcellular localization, or downstream signaling activation, which makes circRNAs important bridges between RNA-level regulation and protein functional modulation. In addition, m6A modification may influence circRNA biogenesis, stability, localization, and functional output, suggesting that circRNAs themselves may be regulated by the epitranscriptome and further participate in OA-related inflammation and tissue injury. Notably, exosome-derived circRNAs can transmit regulatory signals among bone marrow mesenchymal stem cells, chondrocytes (BMSCs), synovial cells, and immune cells, thereby contributing to cross-cellular communication networks. Therefore, circRNAs may jointly regulate OA progression through miRNA sponge activity, protein interactions, m6A modification, and exosomal delivery. This multilayered regulatory feature helps explain why circRNAs can exert either pathogenic or protective effects depending on disease stage and cell type. Future studies are still required to further clarify the specific roles of circRNAs in different cell types and disease stages and to promote their clinical translation in OA diagnosis and targeted therapy.

#### 3.3.3 miRNA

MicroRNAs (miRNAs) are among the most extensively studied ncRNAs in OA and play important roles in processes such as inflammatory responses, cartilage degeneration, cell fate regulation, and tissue repair (Swingler et al., 2019; Duan et al., 2020). Existing studies indicate that miRNAs regulate signaling pathways such as NF-κB, PI3K/Akt, MAPK, and Toll-like receptor 4/myeloid differentiation primary response 88 (TLR4/MyD88), thereby influencing extracellular matrix (ECM) metabolism, apoptosis, autophagy, and senescence in chondrocytes and contributing to the initiation and progression of OA (Liu et al., 2018; Ding et al., 2019; Wang et al., 2020).

In the regulation of inflammation, multiple miRNAs exhibit either anti-inflammatory or pro-inflammatory effects. Ko et al. reported that *miR-128a* is associated with circadian rhythm disruption in OA (Ko et al., 2024). Zhu et al. demonstrated that *miR-29a-3p* suppresses inflammatory responses and delays OA progression through the phosphatase and tensin homolog/phosphoinositide 3-kinase/protein kinase B (PTEN/PI3K/AKT) pathway (Zhu et al., 2024b). Cai et al. reported that *miR-515-5p* exerts anti-inflammatory and anti-apoptotic effects through the TLR4/MyD88/NF-κB signaling axis (Cai et al., 2024). In addition, *miR-99a-5p* negatively regulates Toll-like receptor 8 (TLR8)-mediated innate immune responses (Zhang et al., 2024a), and *miR-8485* attenuates IL-1β-induced inflammatory injury by suppressing cytokine receptor-like factor 1 (CRLF1) and subsequently downregulating MAPK/ERK and PI3K/AKT signaling pathways (Liu et al., 2025d). These findings indicate that miRNAs serve as important molecular hubs linking inflammatory stimuli with abnormal chondrocyte responses.

In cartilage degeneration and ECM homeostasis, miRNAs primarily maintain cartilage integrity by regulating genes associated with matrix synthesis and degradation. *miR-335-5p* has been shown to inhibit endochondral ossification and delay disease progression in temporomandibular joint OA (Xia et al., 2024b). *miR-122-5p* participates in the protective effects of semi-synthetic chondroitin sulfate (Li et al., 2024b), and *miR-92a-3p*-inspired short hairpin RNA (shRNA) promotes chondrogenesis by suppressing SMAD6/7 signaling (Zheng et al., 2024). Meanwhile, *miR-29a* delivered by mesenchymal stem cell (MSC)-derived vesicles also contributes to ECM remodeling (Yang et al., 2024b). Conversely, *miR-6779* is upregulated in OA cartilage and promotes apoptosis of senescent chondrocytes by inhibiting X-linked inhibitor of apoptosis protein (XIAP), thereby exacerbating ECM loss (Li et al., 2025b). Thus, different miRNAs exert bidirectional regulatory roles in OA, functioning either protectively or pathogenically, depending on the context.

miRNAs are also widely involved in regulating chondrocyte fate, including autophagy, apoptosis, pyroptosis, and senescence. Tian et al. found that *miR-145* enhances autophagy and exerts protective effects by downregulating fibroblast growth factor receptor substrate 2 (FRS2) (Tian et al., 2024). In contrast, *miR-34a-5p* accelerates OA progression by inhibiting SESN2-mediated autophagy (Wang et al., 2024b). Inhibition of *miR-103-3p* activates the cytoplasmic polyadenylation element binding protein 3/phosphoinositide 3-kinase/protein kinase B/mammalian target of rapamycin (CPEB3/PI3K/Akt/mTOR) pathway, enhancing autophagy and reducing apoptosis and ECM degradation (Li et al., 2025a). Furthermore, the EZH2/*miR-142-3p*/HMGB1 axis has been shown to participate in the regulation of pyroptosis (Chen et al., 2025), and *miR-653-5p* has also been identified as an important regulator of chondrocyte senescence (Lin et al., 2024). These studies suggest that miRNAs not only influence inflammation and matrix metabolism but also play crucial roles in the dysregulation of chondrocyte fate in OA.

In recent years, circRNA/lncRNA-miRNA-mRNA regulatory networks have emerged as a major focus in OA research. Accumulating evidence suggests that circRNAs and lncRNAs can act as ceRNAs to sponge miRNAs, thereby regulating inflammation, apoptosis, ferroptosis, and ECM metabolism. For example, the PIEZO1/*miR-155-5p*/growth differentiation factor 6 (GDF6)/SMAD2/3 axis reveals the molecular basis of mechanical stress-induced cartilage degeneration (Qin et al., 2024). Similarly, regulatory axes such as *CircTRIM25*/*miR-138-5p*/CREB1 and *MALAT1*/*miR-212-5p*/MyD88 are closely associated with inflammatory amplification and matrix degradation (He et al., 2024). In contrast, networks such as *MEG3*/*miR-885-5p*/SLC7A11 exhibit protective effects. These findings indicate that miRNAs do not act in isolation but are embedded within multilayered ncRNA regulatory networks.

Exosomal miRNAs further expand the translational potential of miRNAs. Exosomal *miR-26b-5p* derived from M2 macrophages can regulate macrophage polarization and inhibit chondrocyte hypertrophy (Qian et al., 2024b). In contrast, *miR-363* derived from M1 macrophages promotes apoptosis and ECM degradation (Si et al., 2024). Exosomes derived from MSCs and bone marrow mesenchymal stem cells (BMSCs) are enriched with miRNAs such as *miR-140*, *miR-146a*, and *miR-21*, which exert anti-inflammatory, anti-apoptotic, and pro-repair effects (Cheng et al., 2024; Scalzone et al., 2024). Moreover, engineered exosomes delivering *miR-146a* or *miR-140* have also shown promising therapeutic potential (Cao et al., 2024a; Liu et al., 2025d).

**FIGURE 3:**
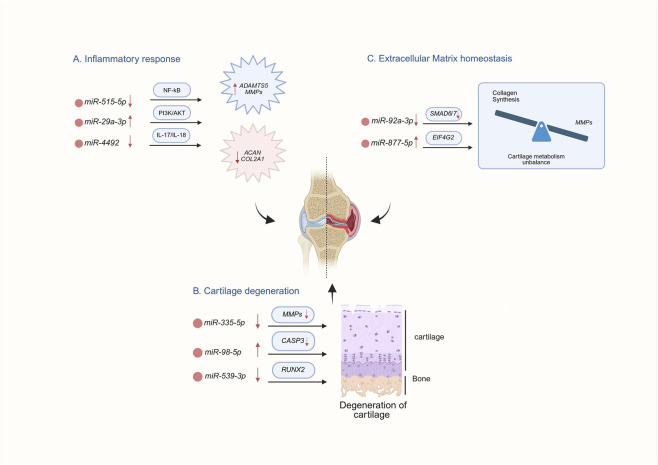
Regulatory roles of miRNAs in OA pathogenesis. miRNAs regulate gene expression and contribute to OA progression by modulating inflammation, chondrocyte function, and ECM homeostasis **(A)** Inflammatory response. miRNAs regulate key inflammatory pathways, including NF-κB, PI3K/AKT, and interleukin signaling. For example, *miR-515-5p, miR-29a-3p,* and *miR-4492* modulate matrix-degrading enzymes, such as ADAMTS5 and MMPs, as well as cartilage matrix genes, such as *ACAN* and *COL2A1*, thereby influencing the inflammatory microenvironment **(B)** Cartilage degeneration. miRNAs participate in cartilage degeneration by regulating apoptosis, differentiation, and matrix degradation. For instance, *miR-335-5p* regulates *MMPs* to affect ECM degradation, *miR-98-5p* targets *CASP3* to modulate apoptosis, and *miR-539-3p* regulates *RUNX2* to influence chondrocyte differentiation **(C)** ECM homeostasis. miRNAs maintain ECM balance by targeting genes such as *SMAD6, SMAD7*, and *EIF4G2.* For example, *miR-92a-3p* and *miR-877-5p* contribute to ECM homeostasis, whereas their dysregulation disrupts the balance between collagen synthesis and matrix degradation. Overall, miRNAs regulate inflammatory signaling, chondrocyte function, and ECM metabolism, thereby promoting cartilage degeneration and OA progression. Created in BioRender. jin, Y. (2026) https://BioRender.com/rz2vam2.

Overall, miRNAs are involved in multiple aspects of OA, including inflammation regulation, cartilage degeneration, dysregulation of cell fate, and tissue repair, and together with lncRNAs, circRNAs, and exosomes form a complex regulatory network (Figure 3). Mechanistically, miRNAs can act as post-transcriptional regulatory hubs by targeting multiple mRNAs and modulating their stability or translational efficiency, thereby linking inflammatory stimulation, mechanical stress, abnormal ECM metabolism, and cell fate transitions. In the OA microenvironment, stimuli such as IL-1β, TNF-α, abnormal mechanical loading, and oxidative stress can alter miRNA expression profiles, whereas dysregulated miRNAs further regulate key pathways, including NF-κB, MAPK, PI3K/Akt, and Wnt/β-catenin signaling. Through these mechanisms, miRNAs influence inflammatory cytokine release, matrix-degrading enzyme expression, chondrocyte apoptosis, senescence, pyroptosis, autophagy, and chondrogenic capacity. Therefore, miRNAs do not merely regulate individual target genes in isolation; instead, they participate in shaping OA progression through coordinated regulation of multiple targets, pathways, and cellular processes. Future studies should focus on representative core miRNAs to clarify their spatiotemporal specificity and clinical translational potential, thereby promoting the transition of miRNA research from mechanistic studies to precision diagnosis and therapeutic applications in OA.

Collectively, lncRNAs, circRNAs, and miRNAs should not be interpreted as three independent classes of regulators in OA, but rather as interconnected components of multilayered post-transcriptional and epigenetic regulatory networks. In ceRNA networks, lncRNAs and circRNAs can act as molecular sponges for specific miRNAs, thereby relieving miRNA-mediated repression of target mRNAs and modulating the expression of genes involved in inflammatory signaling, ECM degradation, chondrocyte apoptosis, senescence, pyroptosis, ferroptosis, and cartilage repair. Meanwhile, miRNAs serve as central regulatory hubs that integrate upstream signals from inflammatory cytokines, mechanical stress, oxidative stress, and metabolic disturbance and translate them into downstream changes in gene expression. In addition, extracellular vesicles can transfer lncRNAs, circRNAs, and miRNAs among chondrocytes, synovial cells, subchondral bone cells, mesenchymal stem cells, and immune cells, thereby extending ncRNA-mediated regulation from intracellular gene control to intercellular and inter-tissue communication. Therefore, ncRNA-mediated regulation in OA is better understood as an integrated network connecting inflammatory activation, mechanical stress responses, ECM metabolism, cell fate transitions, and tissue repair. Future studies should move beyond single ncRNA–single target models and focus on identifying core ncRNA regulatory modules, disease-stage-specific ceRNA networks, and spatially resolved intercellular communication pathways in OA.

### 3.4 Chromatin accessibility, enhancers and super-enhancers in OA

Chromatin accessibility represents an important layer of epigenomic regulation and reflects whether specific genomic regions are open and permissive for the binding of transcription factors and transcriptional regulatory complexes (Klemm et al., 2019). Unlike DNA methylation, histone modifications, and ncRNAs, chromatin accessibility more directly indicates the functional state of gene regulatory regions (Liu et al., 2018; Klemm et al., 2019). Open chromatin regions are commonly enriched at promoters, enhancers, and other cis-regulatory elements and are closely associated with transcriptional activity (Liu et al., 2018; Klemm et al., 2019; Roberts and Rice, 2024). Therefore, chromatin accessibility can be considered an important bridge linking environmental stimuli, epigenetic modifications, and transcriptional output (Roberts and Rice, 2024; Kramer et al., 2025). In the context of OA, inflammatory stimulation, mechanical stress, oxidative stress, and aging may reshape the transcriptional programs of chondrocytes and other joint-resident cells by altering chromatin accessibility (Hatzikotoulas et al., 2025; Kramer et al., 2025).

During OA progression, joint-resident cells are chronically exposed to pro-inflammatory cytokines and abnormal mechanical environments (Liu et al., 2018; Fazio et al., 2024). These stimuli may induce dynamic alterations in chromatin architecture, making genomic regions associated with inflammation, ECM degradation, cellular senescence, and hypertrophic differentiation more accessible to transcription factors (Roberts and Rice, 2024; Kramer et al., 2025). For example, transcription factors such as NF-κB, AP-1, RUNX2, and STAT family members not only participate in OA-related signaling cascades but may also bind to accessible chromatin regions and drive the expression of inflammatory mediators, chemokines, and matrix-degrading enzymes (Neefjes et al., 2020; Fazio et al., 2024). Compared with transient activation of signaling pathways, changes in chromatin accessibility may exert more durable regulatory effects, thereby maintaining chondrocytes in a pro-inflammatory, catabolic, or degenerative phenotype (Larsen et al., 2021; Roberts and Rice, 2024; Kramer et al., 2025). This process may help explain the persistence of pathological transcriptional programs in OA, even after the initial stimuli are reduced (Larsen et al., 2021; Kramer et al., 2025).

Enhancers are critical cis-regulatory elements that control cell type-specific gene expression, and their activity is closely associated with open chromatin states and histone marks such as H3K27ac and H3K4me1 (Shlyueva et al., 2014). In OA, enhancer remodeling may represent an important mechanism underlying inflammatory activation and ECM imbalance (Liu et al., 2018; Roberts and Rice, 2024). Persistent stimulation by IL-1β or TNF-α may recruit inflammation-related transcription factors to specific enhancer regions and increase local histone acetylation, thereby enhancing the transcriptional activity of inflammatory and catabolic genes (Neefjes et al., 2020). Meanwhile, regulatory regions associated with the maintenance of chondrocyte homeostasis may become less accessible or acquire repressive histone modifications, leading to reduced expression of cartilage matrix-related genes such as *SOX9*, *COL2A1*, and *ACAN* (Bittner et al., 2024; Roberts and Rice, 2024; Kramer et al., 2025). Thus, epigenetic dysregulation in OA should not be viewed simply as alterations in individual promoters or single genes, but rather as a broader remodeling of the enhancer landscape (Liu et al., 2018; Bittner et al., 2024; Roberts and Rice, 2024; Kramer et al., 2025).

Super-enhancers are large clusters of adjacent enhancers that are densely occupied by transcription factors, co-activators, Mediator, and strong H3K27ac signals (Hnisz et al., 2013; Whyte et al., 2013). They often drive high-level expression of genes that determine cell identity or disease-associated phenotypes (Hnisz et al., 2013). Although studies specifically investigating super-enhancers in OA remain limited, enhancer remodeling and altered chromatin accessibility have been increasingly recognized as important regulatory features in OA cartilage, suggesting that super-enhancer remodeling may theoretically contribute to the maintenance of pathological transcriptional states in chondrocytes (Roberts and Rice, 2024; Kramer et al., 2025). Under inflammatory, senescent, or abnormal mechanical conditions, aberrantly activated super-enhancer regions may form near genes involved in inflammatory responses, matrix degradation, cellular senescence, or hypertrophic differentiation, thereby amplifying pathological signals and stabilizing OA-associated gene expression programs (Brown et al., 2014; Tasdemir et al., 2016). Conversely, reduced super-enhancer activity at key regulatory regions required for maintaining the normal chondrocyte phenotype may contribute to the loss of cartilage-protective functions (Tasdemir et al., 2016).

More broadly, chromatin accessibility and enhancer remodeling may also provide a mechanistic link between genetic susceptibility and OA phenotypes (Rice et al., 2020; Roberts and Rice, 2024). Many disease-associated genetic variants are located outside protein-coding regions and are enriched in non-coding regulatory elements (Roberts and Rice, 2024; Hatzikotoulas et al., 2025). These variants may influence transcription factor binding, enhancer activity, or enhancer-promoter interactions, thereby altering the expression of OA-related genes (Thulson et al., 2022; Bittner et al., 2024; Kramer et al., 2025). Therefore, integrating genome-wide association studies, DNA methylomes, histone modification profiles, ATAC-seq data, and transcriptomic datasets may help identify functionally relevant regulatory elements and clarify how genetic risk is translated into disease phenotypes through epigenetic mechanisms (Thulson et al., 2022; Bittner et al., 2024; Hatzikotoulas et al., 2025; Kramer et al., 2025).

At present, research on chromatin accessibility, enhancers, and super-enhancers in OA is still in its early stages. Future studies should apply technologies such as ATAC-seq, ChIP-seq, CUT&Tag, CUT&RUN, single-cell ATAC-seq, and spatial multi-omics to systematically characterize chromatin regulatory landscapes across different joint tissues, cell types, and disease stages. In particular, integrative analyses of open chromatin regions, histone modifications, transcription factor binding, and gene expression changes may shift the field from a “single-gene alteration” model toward a “regulatory element network remodeling” framework. Such advances will not only deepen our understanding of epigenetic mechanisms in OA, but also facilitate the identification of novel biomarkers for disease stratification and precision therapeutic targets.

### 3.5 Three-dimensional genome organization in OA

Three-dimensional genome organization has emerged as an important field in epigenomic research, focusing on how genomic DNA is spatially folded within the nucleus and how this organization influences gene regulation (Xia et al., 2024a; Beliveau and Akilesh, 2025). Traditional epigenetic studies have mainly focused on DNA methylation, histone modifications, and non-coding RNA-mediated regulation along the linear genome. In contrast, three-dimensional genome studies further reveal how distal regulatory elements interact with target genes through spatial proximity (Uyehara and Apostolou, 2023; Papantonis and Oudelaar, 2025). The genome is not randomly distributed in the nucleus, but is organized into hierarchical structures, including chromatin loops, topologically associating domains (TADs), chromatin compartments, and enhancer-promoter interactions (Dixon et al., 2012; Xia et al., 2024a). These structures influence the recruitment of transcription factors, co-activators, RNA polymerase II, and other transcriptional regulatory complexes, thereby fine-tuning gene expression ](Uyehara and Apostolou, 2023; Xia et al., 2024a; Papantonis and Oudelaar, 2025).

In OA, abnormal expression of genes involved in inflammatory responses, ECM degradation, chondrocyte hypertrophic differentiation, and cellular senescence may not be fully explained by epigenetic alterations at their own promoter regions (Bittner et al., 2024; Roberts and Rice, 2024). Distal enhancers or other cis-regulatory elements may spatially contact target gene promoters through chromatin looping and thereby regulate the transcriptional activity of OA-related genes (Bittner et al., 2024). For example, under inflammatory stimulation or abnormal mechanical loading, transcription factors such as NF-κB, AP-1, and RUNX2 may bind to distal enhancer regions and promote the expression of matrix-degrading enzymes, chemokines, or inflammatory mediators through enhancer-promoter interactions (Barter et al., 2021; Nagata et al., 2022). Conversely, reduced spatial communication between cartilage-protective genes and their distal regulatory elements may contribute to the downregulation of genes required for chondrocyte homeostasis (Nagata et al., 2022; Bittner et al., 2024). Therefore, abnormal three-dimensional genome organization may represent an important mechanism underlying the remodeling of gene regulatory networks in OA (Bittner et al., 2024).

TADs are basic functional units of three-dimensional genome organization and restrict the range of enhancer-promoter interactions, thereby maintaining regulatory specificity (Lupiáñez et al., 2015). When TAD boundaries are disrupted or chromatin architecture is reorganized, enhancers may aberrantly activate neighboring or inappropriate target genes, leading to disease-associated transcriptional programs (Lupiáñez et al., 2015). In the context of OA, chronic inflammation, cellular senescence, and mechanical stress may alter chromatin conformation and allow inflammatory, catabolic, or senescence-associated genes to receive abnormal distal regulatory inputs (Barter et al., 2021; Thulson et al., 2022; Bittner et al., 2024). At the same time, genes involved in maintaining the chondrocyte phenotype may lose normal enhancer support due to disrupted spatial regulatory structures (Thulson et al., 2022; Bittner et al., 2024). Such alterations at the level of three-dimensional genome organization may help explain why OA cells can establish relatively stable pathological transcriptional states after prolonged stimulation (Barter et al., 2021; Bittner et al., 2024).

Three-dimensional genome organization may also provide a mechanistic link between genetic susceptibility and OA phenotypes (Thulson et al., 2022; Bittner et al., 2024). Many OA-associated genetic variants are located in non-coding regions and do not directly alter protein-coding sequences (Peña-Martínez and Rodríguez-Martínez, 2024; Roberts and Rice, 2024). Instead, they may influence the activity of enhancers, insulators, or other regulatory elements (Peña-Martínez and Rodríguez-Martínez, 2024; Roberts and Rice, 2024). Through three-dimensional genome interactions, these non-coding variants can regulate target gene expression over long genomic distances (Thulson et al., 2022; Bittner et al., 2024; Kramer et al., 2025). For example, a risk variant located within a distal enhancer may alter transcription factor binding or chromatin loop formation, thereby affecting genes involved in cartilage homeostasis, inflammatory responses, or osteochondral crosstalk (Peña-Martínez and Rodríguez-Martínez, 2024; Roberts and Rice, 2024; Kramer et al., 2025). Therefore, integrating genome-wide association studies with chromatin accessibility profiling, histone modification maps, and three-dimensional genome datasets may help identify the true functional target genes of OA risk loci (Thulson et al., 2022; Bittner et al., 2024).

At present, studies on three-dimensional genome organization in OA remain limited (Thulson et al., 2022; Bittner et al., 2024). Future research may apply technologies such as high-throughput chromosome conformation capture (Hi-C), Capture-C, high-throughput chromosome conformation capture combined with chromatin immunoprecipitation (HiChIP), and proximity ligation-assisted chromatin immunoprecipitation sequencing (PLAC-seq) to systematically characterize chromatin interaction landscapes in OA chondrocytes, subchondral bone cells, and synovial cells (Juric et al., 2019; Bittner et al., 2024; Roberts and Rice, 2024). In addition, combining these approaches with single-cell sequencing, single-cell ATAC-seq, and spatial omics technologies may further reveal cell type-specific and stage-specific three-dimensional chromatin regulatory patterns (Fan et al., 2024b). In particular, integrating three-dimensional genome organization with DNA methylation, histone modifications, ncRNAs, and transcriptomic data will help establish a comprehensive regulatory framework linking genetic variation, epigenetic remodeling, and disease phenotypes (Thulson et al., 2022; Bittner et al., 2024; Liu et al., 2025d).

Overall, three-dimensional genome organization provides a new perspective for understanding epigenetic regulation in OA. It suggests that abnormal gene expression in OA is not only the result of promoter methylation or histone modification changes at individual genes, but may also arise from the global remodeling of distal regulatory elements, enhancer-promoter interactions, TAD structures, and nuclear chromatin architecture. With the development of three-dimensional genomics and single-cell multi-omics technologies, this field may provide new theoretical foundations for OA molecular stratification, functional interpretation of genetic risk loci, and the discovery of precision therapeutic targets.

### 3.6 Tissue-specific epigenetic regulation across joint tissues in OA

OA is not merely a disease of articular cartilage, but a complex degenerative disorder affecting the whole joint organ (Loeser et al., 2012). In addition to articular cartilage, subchondral bone, synovium, meniscus, ligaments, and adipose tissues are all involved in OA initiation and progression (Loeser et al., 2012; Sanchez-Lopez et al., 2022). Different joint tissues have distinct cellular compositions, mechanical environments, metabolic states, and inflammatory profiles; therefore, their epigenetic alterations are likely to be highly tissue-specific (Li et al., 2024a; Kalliolias et al., 2025; Raut et al., 2025a). Restricting OA epigenetic studies to chondrocytes or a single tissue type may not fully explain disease heterogeneity and progression (Kalliolias et al., 2025; Raut et al., 2025a). Thus, comparing epigenetic regulatory features across different joint tissues from a whole-joint perspective is essential for understanding the systemic pathological mechanisms of OA and identifying tissue-specific therapeutic targets (Kalliolias et al., 2025; Raut et al., 2025a).

In articular cartilage, epigenetic regulation primarily affects chondrocyte phenotype maintenance, ECM homeostasis, and cartilage degeneration (Lafont et al., 2023; Hatzikotoulas et al., 2025). Under physiological conditions, chondrocytes maintain cartilage matrix synthesis through the stable expression of genes such as *SOX9*, *COL2A1*, and *ACAN* (Neefjes et al., 2020; Michelacci et al., 2023). Under OA conditions, however, inflammatory cytokines, mechanical stress, and oxidative stress can induce aberrant DNA methylation, histone modification remodeling, and altered ncRNA expression, thereby suppressing anabolic cartilage genes and activating catabolic genes such as *MMP13* and *ADAMTS5* (Lafont et al., 2023; Hatzikotoulas et al., 2025). In addition, chondrocytes may undergo hypertrophic-like differentiation, senescence, apoptosis, pyroptosis, and ferroptosis, all of which are closely associated with epigenetic regulation (Michelacci et al., 2023; Guan et al., 2024; Huang and Cai, 2025). Therefore, epigenetic dysregulation in cartilage mainly reflects the transition of chondrocytes from a homeostatic state toward inflammatory, catabolic, and degenerative phenotypes (Lafont et al., 2023; Michelacci et al., 2023; Hatzikotoulas et al., 2025).

Subchondral bone is another key tissue involved in OA progression (Hu et al., 2021; Chen et al., 2024d). Its pathological alterations include enhanced bone remodeling, changes in trabecular architecture, subchondral sclerosis, vascular and nerve invasion, and osteophyte formation (Hu et al., 2021). Unlike cartilage, epigenetic regulation in subchondral bone may be more closely associated with the differentiation and function of osteoblasts, osteoclasts, osteocytes, and BMSCs(Jeffries et al., 2016; Park-Min, 2017; Chen et al., 2024d). For example, DNA methylation and histone modifications may regulate genes involved in osteogenic differentiation, osteoclast activity, angiogenesis, and osteochondral crosstalk, thereby influencing subchondral bone remodeling (Jeffries et al., 2016; Park-Min, 2017). Abnormal subchondral bone not only alters the local biomechanical environment of the joint, but also affects overlying cartilage through cytokines, extracellular vesicles, and metabolites (Chen et al., 2024b; Liu et al., 2025a). Thus, epigenetic changes in subchondral bone may serve as a link among mechanical stress, metabolic alterations, and inflammatory signaling in OA (Jeffries et al., 2016; Chen et al., 2024d; Liu et al., 2025a).

Synovial tissue also plays an important role in inflammatory amplification and pain generation in OA (Philpott et al., 2025). Although synovitis in OA is usually less intense than that in rheumatoid arthritis, low-grade chronic synovial inflammation can continuously release IL-1β, TNF-α, IL-6, chemokines, and matrix-degrading molecules, thereby aggravating cartilage damage and pain (Damerau et al., 2024; Philpott et al., 2025). Epigenetic abnormalities in synovial fibroblasts, macrophages, and other immune cells may promote sustained activation of inflammatory genes (Damerau et al., 2024; Roberts et al., 2025; Laouteouet et al., 2026). For instance, pro-inflammatory stimulation may alter chromatin accessibility and histone acetylation at inflammation-related loci in synovial cells, resulting in persistent and amplified inflammatory responses (Roberts et al., 2025). Meanwhile, ncRNAs and DNA methylation may also regulate synovial cell activation, immune cell infiltration, and cytokine secretion (Nau et al., 2025). Therefore, epigenetic regulation in synovium is more closely associated with the maintenance of the inflammatory microenvironment and aberrant inter-tissue communication (Liu et al., 2024c; Roberts et al., 2025; Laouteouet et al., 2026).

The meniscus and periarticular adipose tissues are also emerging tissues of interest in OA epigenetic research. Meniscal injury or degeneration can markedly alter joint load distribution and further promote cartilage degeneration (Ozeki et al., 2022; Hurmuz et al., 2024). Under mechanical stress and inflammatory stimulation, meniscal cells may undergo epigenetic changes similar to those observed in chondrocytes, including activation of matrix-degrading genes, increased inflammatory mediator expression, and enhanced cellular senescence (Stone et al., 2014; Swahn et al., 2023). Adipose tissues, particularly the infrapatellar fat pad in the knee joint, can secrete adipokines, inflammatory mediators, and extracellular vesicles that influence synovial inflammation, cartilage metabolism, and pain sensitivity (Cao Y. et al., 2024; Mallia et al., 2025). Epigenetic regulation in adipose tissues may therefore be associated with metabolic inflammation, immune cell infiltration, and amplification of the local inflammatory response (Roberts et al., 2025). Thus, the meniscus and adipose tissues should not be considered merely as passively affected tissues in OA, but rather as active contributors to disease progression through tissue-specific epigenetic alterations (Ozeki et al., 2022; Cao et al., 2024b; Mallia et al., 2025).

Epigenetic alterations in different joint tissues do not occur in isolation, but may form cross-tissue regulatory networks through cytokines, extracellular vesicles, ncRNAs, and metabolites (Wang et al., 2022; Liu et al., 2025a; Raut et al., 2025b; Roberts et al., 2025; Sun et al., 2025). For example, signals derived from subchondral bone can influence chondrocyte metabolism (Liu et al., 2025a), synovium-derived inflammatory cytokines can accelerate matrix degradation (Chou et al., 2020), and damage-associated molecules released by chondrocytes can in turn activate synovial inflammation (Chou et al., 2020). This inter-tissue crosstalk suggests that OA epigenetic research should move beyond a single-cell-type framework and integrate multilayered information from cartilage, subchondral bone, synovium, and other joint tissues (Wang et al., 2022; Raut et al., 2025b; Roberts et al., 2025). In particular, extracellular vesicle-mediated transfer of miRNAs, lncRNAs, or circRNAs may represent an important mechanism by which epigenetic regulatory signals are transmitted among different tissues (Liu et al., 2025a; Sun et al., 2025; Yan et al., 2026).

At present, tissue-specific epigenetic research in OA still faces several challenges (Hatzikotoulas et al., 2025; Wilson et al., 2025). First, many studies use bulk cartilage or mixed tissue samples, making it difficult to distinguish cell type-specific epigenetic alterations (Huang et al., 2022; Raut et al., 2025b). Second, differences in joint sites, disease stages, and patient characteristics may contribute to inconsistent findings (Wilson et al., 2025). Third, epigenetic studies of subchondral bone, synovium, meniscus, and adipose tissues remain less extensive than those of cartilage (Fan et al., 2024a; Hatzikotoulas et al., 2025). Future studies should combine single-cell RNA sequencing, single-cell ATAC-seq, spatial transcriptomics, spatial epigenomics, and multi-omics integration to systematically map the epigenetic landscapes of different OA tissues and cell populations (Fan et al., 2024a; Liu et al., 2025d; Raut et al., 2025b). Such approaches will help identify tissue-specific regulatory networks, explain OA heterogeneity, and provide new targets for precision therapy (Hatzikotoulas et al., 2025; Liu et al., 2025d; Raut et al., 2025b; Wilson et al., 2025).

Overall, epigenetic regulation in OA exhibits marked tissue specificity. Epigenetic alterations in cartilage mainly affect matrix homeostasis and chondrocyte phenotype; those in subchondral bone are more closely related to bone remodeling and osteochondral crosstalk; those in synovium primarily promote persistent inflammation and immune microenvironment remodeling; and those in the meniscus and adipose tissues may contribute to OA progression through mechanical and metabolic-inflammatory pathways. Future integration of epigenetic information at the whole-joint, multi-tissue, and single-cell levels will help advance OA research from single-tissue mechanisms toward a systemic disease network.

### 3.7 Integration of genetics, epigenomics, single-cell and spatial multi-omics in OA

With the rapid development of high-throughput sequencing and computational biology, OA research has gradually shifted from the analysis of individual candidate genes or isolated signaling pathways toward the systematic integration of genetics, epigenomics, transcriptomics, proteomics, metabolomics, single-cell omics, and spatial omics (Liu et al., 2025d; Wei et al., 2025; Wilson et al., 2025; Sharma, 2026). OA is a highly heterogeneous disease influenced by genetic susceptibility, mechanical loading, inflammation, aging, metabolic abnormalities, and environmental exposures](Roberts et al., 2025; Wilson et al., 2025). Although single-omics studies can reveal molecular alterations at a specific level, they are often insufficient to fully explain tissue-specific pathological changes, cellular state transitions, and inter-individual variability (Raut et al., 2025b). Therefore, multi-omics integration may help establish a continuous regulatory framework linking genetic risk, epigenetic remodeling, cellular dysfunction, and tissue pathological phenotypes (Liu et al., 2025d; Wei et al., 2025).

Genetic studies have provided important clues regarding OA susceptibility (Boer et al., 2021; Hatzikotoulas et al., 2025). Genome-wide association studies have identified multiple OA risk loci, but many risk variants are located in non-coding regions and do not directly alter protein-coding sequences (Boer et al., 2021; Arruda et al., 2024; Roberts and Rice, 2024; Hatzikotoulas et al., 2025). This suggests that genetic risk may act mainly through regulatory elements such as enhancers, promoters, and insulators (Arruda et al., 2024; Bittner et al., 2024; Roberts and Rice, 2024). Integrating genetic data with DNA methylomes, chromatin accessibility profiles, histone modification maps, and three-dimensional genome datasets may help determine whether risk loci overlap with functional regulatory regions and further identify their true target genes and pathological pathways (Arruda et al., 2024; Bittner et al., 2024; Hatzikotoulas et al., 2025; Tian et al., 2025).

Single-cell and spatial omics provide new tools for resolving OA heterogeneity (Fan et al., 2024b; Raut et al., 2025b). Traditional bulk RNA sequencing or bulk epigenomic profiling reflects average molecular changes at the tissue level and may obscure key regulatory events in specific cell subpopulations (Huang et al., 2025; Raut et al., 2025b). Single-cell RNA sequencing and single-cell ATAC-seq can reveal the transcriptional states and chromatin regulatory features of distinct cell populations, including homeostatic chondrocytes, inflammatory chondrocytes, hypertrophic-like chondrocytes, senescent-like chondrocytes, synovial fibroblasts, macrophages, and subchondral bone-associated cells (Liu et al., 2018; Kramer et al., 2025). By jointly analyzing gene expression and chromatin accessibility, these approaches can further infer key transcription factors, enhancers, and regulatory networks driving cellular state transitions (Liu et al., 2018; Kramer et al., 2025).

Spatial omics further complements single-cell technologies by preserving tissue localization (Fan et al., 2024a; Xie et al., 2026). OA joint tissues have distinct spatial organization (Fan et al., 2024a; Raut et al., 2025b). For example, the superficial, middle, deep, and calcified zones of cartilage differ in mechanical loading, nutrient diffusion, cellular morphology, and matrix composition (Sophia Fox et al., 2009; Fan et al., 2024a). Synovial inflammatory regions, cartilage lesion margins, and subchondral bone remodeling areas also exhibit distinct local microenvironments (Hu et al., 2021; Fan et al., 2024a). Spatial transcriptomics, spatial proteomics, and spatial epigenomics enable the analysis of cell distribution, gene expression, and regulatory states while preserving tissue architecture, thereby helping to clarify local lesion formation, inflammation-cartilage interactions, and osteochondral crosstalk in OA (Fan et al., 2024a; Delos Santos et al., 2026).

Proteomics and metabolomics can bridge transcriptional alterations and functional phenotypes (Liu et al., 2025d; Wei et al., 2025). Because mRNA levels do not always correspond to protein abundance, enzyme activity, or metabolic flux, proteomics provides a more direct view of pathway activation, ECM remodeling, and cellular functional states (Rai et al., 2024). Metabolomics can reveal alterations in energy metabolism, oxidative stress, lipid metabolism, amino acid metabolism, and ferroptosis-related pathways (Wang et al., 2025a; Wei et al., 2025). Importantly, metabolites can also serve as substrates or cofactors for epigenetic modifications (Dai et al., 2020). For example, S-adenosylmethionine, acetyl-CoA, α-ketoglutarate, lactate, NAD+, and malonyl-CoA participate in DNA methylation, histone acetylation, demethylation, lactylation, malonylation, and deacetylation (Dai et al., 2020). Thus, metabolic reprogramming and epigenetic regulation are closely interconnected (Dai et al., 2020; Wang et al., 2025a).

Multi-omics integration may also promote molecular stratification and precision therapy in OA (Liu et al., 2025d). Different patients may exhibit inflammation-dominant, cartilage matrix degradation-dominant, subchondral bone remodeling-dominant, senescence-associated, or metabolism-related OA phenotypes (Sharma, 2026). Integrating genomics, epigenomics, single-cell omics, spatial omics, proteomics, metabolomics, and clinical imaging data may help identify molecular OA subtypes more accurately and guide individualized therapeutic strategies (Liu et al., 2025d; Sharma, 2026). However, current multi-omics studies still face challenges, including limited sample sizes, platform differences, batch effects, analytical complexity, and insufficient functional validation (Wei et al., 2025; Sharma, 2026). Future research should establish large-scale, multicenter, longitudinal OA multi-omics cohorts and combine epigenome editing, organoid systems, animal models, and human tissues to validate the causal roles of key regulatory networks (Lyu et al., 2025).

Overall, the integration of genetics, epigenomics, single-cell omics, and spatial multi-omics provides a systematic framework for understanding the complex pathogenesis of OA. Future studies should move beyond individual genes or single epigenetic marks toward an integrated model of “genetic variation–regulatory elements–epigenetic states–cell type-specific transcriptional programs–spatial microenvironment–tissue pathological phenotypes,” thereby advancing OA epigenetic research toward precision stratification and disease-modifying therapy.

## 4 Translational implications and therapeutic potential

With the growing understanding of epigenetic regulatory mechanisms in OA, alterations in DNA methylation, histone modifications, ncRNAs, chromatin accessibility, and three-dimensional genome organization not only provide new insights into disease pathogenesis, but also offer potential opportunities for early diagnosis, disease stratification, and precision therapy. Unlike genetic mutations, epigenetic alterations are dynamic, reversible, and responsive to environmental stimuli. They can reflect the long-term effects of mechanical loading, inflammation, aging, and metabolic abnormalities on joint-resident cells. Therefore, epigenetic regulation may serve as an important bridge between molecular mechanisms and clinical translation in OA.

First, epigenetic biomarkers hold potential value for early diagnosis and disease classification. Current OA diagnosis mainly relies on symptoms, imaging findings, and structural joint damage, which often become evident only after disease progression. In contrast, DNA methylation profiles, circulating ncRNAs, exosomal miRNAs, and tissue-specific chromatin regulatory features may change before obvious structural destruction occurs. Therefore, integrating epigenetic biomarkers with clinical symptoms, imaging characteristics, and multi-omics data may improve early detection and promote the development of molecular classification systems for OA.

Second, the reversibility of epigenetic regulation makes epigenetic regulators attractive therapeutic targets. DNMTs, TET enzymes, HATs, histone deacetylases (HDACs), histone methyltransferases, and histone demethylases can influence chondrocyte metabolism, inflammatory responses, cell death, and matrix degradation by regulating key gene expression programs. Targeting these regulators may help reshape abnormal transcriptional programs, restore chondrocyte homeostasis, and reduce OA-related pathological changes. However, because epigenetic enzymes have broad functions across different tissues and cell types, future therapeutic strategies should emphasize local, cell type-specific, and disease stage-specific regulation.

Non-coding RNAs also provide promising therapeutic opportunities for OA. miRNAs, lncRNAs, and circRNAs participate in OA progression by regulating inflammatory signaling, ECM metabolism, apoptosis, pyroptosis, ferroptosis, and cellular senescence. Based on these mechanisms, RNA-based therapeutic strategies, including miRNA mimics, miRNA inhibitors, antisense oligonucleotides, small interfering RNAs, and engineered circRNAs, may be used to correct dysregulated post-transcriptional networks in OA. Compared with traditional small-molecule drugs, RNA therapeutics offer higher sequence specificity, but their stability, immunogenicity, and tissue delivery efficiency still require further optimization.

In addition, extracellular vesicles, nanoparticles, hydrogels, and other biomaterial-based delivery systems provide new possibilities for epigenetic therapy in OA. Because the joint cavity is relatively enclosed, local delivery may increase drug concentrations within diseased joints while reducing systemic adverse effects. Mesenchymal stem cell-derived extracellular vesicles, engineered extracellular vesicles, and stimuli-responsive nanoplatforms may serve as carriers for miRNAs, siRNAs, antisense oligonucleotides, or small-molecule epigenetic drugs, enabling local regulation of target tissues such as cartilage, synovium, and subchondral bone.

Overall, epigenetics provides a new direction for shifting OA treatment from symptomatic control toward disease modification. In the future, combining molecular stratification, local delivery systems, and multi-omics analysis may allow epigenetic interventions to play an important role in early diagnosis, precision classification, and individualized therapy for OA.

## 5 Current limitations, model heterogeneity and future challenges

Although substantial progress has been made in OA epigenetics in recent years, several important challenges remain. Existing studies have revealed the roles of epigenetic regulation in OA pathogenesis at multiple levels, including DNA methylation, histone modifications, ncRNAs, chromatin accessibility, and three-dimensional genome organization. However, many studies are still largely descriptive or correlative and often focus on individual molecules, isolated pathways, or a single class of epigenetic marks. A systematic regulatory framework capable of explaining OA heterogeneity, disease stage-specific changes, and clinical phenotypic diversity has not yet been fully established.

First, model heterogeneity affects the comparability and reproducibility of findings. Commonly used OA models include surgically induced models, chemically induced models, natural aging models, and *in vitro* cell models stimulated with IL-1β or TNF-α. These models do not represent identical pathological processes. Surgical models mainly reflect mechanical instability and post-traumatic degeneration, aging models more closely resemble chronic age-related changes, whereas cytokine-stimulated models primarily capture acute effects of specific inflammatory signals. Therefore, epigenetic alterations observed in different models may represent distinct pathological contexts and should not be considered directly equivalent. Future studies should clearly define the OA subtype and disease stage represented by each model and strengthen cross-model validation.

Second, sample source and tissue-cell heterogeneity remain major limitations. OA patients differ in age, sex, body mass index, affected joint site, disease severity, inflammatory status, and metabolic profile, all of which may influence epigenetic features. Moreover, many human OA samples are obtained from patients undergoing joint replacement surgery and therefore usually represent end-stage disease, making it difficult to capture early OA and dynamic disease progression. Traditional bulk tissue sequencing also mixes signals from different tissues and cell populations, potentially obscuring key epigenetic changes in specific cell subsets. Future studies should combine standardized clinical stratification, longitudinal cohorts, single-cell omics, and spatial omics to resolve tissue-, cell type-, and spatial region-specific regulatory networks.

Third, insufficient causal validation remains a central issue in OA epigenetic research. Many studies have identified methylation sites, histone-modifying enzymes, ncRNAs, or open chromatin regions associated with OA severity. However, whether these alterations are drivers of disease progression or secondary consequences of inflammation, mechanical injury, and tissue degeneration remains unclear. Future research should strengthen functional validation using CRISPR/dCas9-based epigenome editing, CUT&Tag, CUT&RUN, ChIP-qPCR, ncRNA knockdown or overexpression, organoid models, animal models, and human tissue samples to clarify the causal roles of key epigenetic events.

Fourth, reproducibility and standardization need further improvement. Differences in tissue sampling regions, sample processing methods, sequencing platforms, quality control criteria, and bioinformatics pipelines can lead to inconsistent results. In particular, emerging modifications such as histone lactylation and malonylation are still at an early stage in OA research, and related conclusions require independent cohorts and multi-platform validation. Future studies should emphasize standardized experimental workflows, data sharing, cross-cohort validation, and multicenter research to improve the reliability of OA epigenetic findings.

Fifth, clinical translation still faces challenges related to targeting, delivery efficiency, and safety. Epigenetic regulators often have broad biological functions, and the same regulator may exert different or even opposite effects across tissues, cell types, or disease stages. Systemic intervention targeting DNMTs, histone-modifying enzymes, or ncRNAs may cause off-target effects or adverse reactions. In addition, articular cartilage is avascular and contains a dense ECM, which limits drug penetration into deep cartilage. Therefore, more precise local delivery strategies, such as extracellular vesicles, lipid nanoparticles, hydrogels, biomimetic materials, and inflammation-responsive delivery systems, are needed to improve intra-articular stability, targeting accuracy, and sustained release.

Overall, OA epigenetic research is moving from single-molecule description toward systematic investigation at multi-tissue, multi-cellular, multi-omics, and spatially resolved levels. Future studies should focus on model and sample standardization, integration of single-cell and spatial multi-omics, causal validation of key mechanisms, and development of precision delivery technologies. Only by addressing model heterogeneity, reproducibility, mechanistic causality, and translational challenges can epigenetics become a solid foundation for early diagnosis, molecular stratification, and disease-modifying therapy in OA.

## 6 Conclusion

Epigenetic regulation plays a critical role in the initiation and progression of OA and provides an important conceptual framework for understanding disease heterogeneity, chronic progression, and environmental responsiveness. Mechanical loading, inflammatory stimulation, aging, oxidative stress, and metabolic abnormalities can reshape gene expression programs in joint-resident cells through mechanisms such as DNA methylation, histone modifications, and ncRNAs, thereby influencing key pathological processes including cartilage matrix homeostasis, inflammatory responses, cell death, cellular senescence, subchondral bone remodeling, and synovial inflammation. Therefore, epigenetics not only helps clarify the molecular mechanisms of OA, but also offers new opportunities for early diagnosis, disease stratification, and targeted intervention.

In recent years, OA epigenetic research has expanded from classical mechanisms, including DNA methylation, histone modifications, and non-coding RNA-mediated regulation, to broader layers of epigenomic regulation. Chromatin accessibility, enhancer and super-enhancer remodeling, three-dimensional genome organization, tissue-specific regulation, and the integration of genetics with multi-omics provide new perspectives for explaining aberrant OA-related gene expression, cellular state transitions, inter-tissue crosstalk, and disease heterogeneity. These emerging directions are shifting OA research from the analysis of individual molecules or isolated pathways toward cell type-specific, spatially resolved, and multilayered regulatory network analysis.

Future OA epigenetic studies should further integrate single-cell omics, spatial omics, multi-omics analysis, and functional validation to identify key regulatory networks across different tissues, cell subpopulations, and disease stages. In parallel, epigenome editing, organoid systems, animal models, and human tissue-based studies are needed to validate the causal roles of key mechanisms, while safer and more effective local delivery and precision intervention strategies should be developed. With continued advances in mechanistic research and translational platforms, epigenetic regulation may become an important foundation for early detection, precision stratification, and disease-modifying therapy in OA.

## Glossary

5-hmC: 5-hydroxymethylcytosine
5-mC: 5-methylcytosine
*ACAN*: aggrecan
ADAMTS: a disintegrin and metalloproteinase with thrombospondin motifs
AP-1: activator protein-1
ATAC-seq: assay for transposase-accessible chromatin with sequencing
*BMP7*: bone morphogenetic protein 7
BMSC: bone marrow mesenchymal stem cell
BMSCs: bone marrow mesenchymal stem cells
CASP3: caspase 3
ceRNA: competitive endogenous RNA
ceRNAs: competitive endogenous RNAs
ChIP-seq: chromatin immunoprecipitation sequencing
*COL2A1*: collagen type II alpha 1 chain
CPEB3: cytoplasmic polyadenylation element binding protein 3
CpG: cytosine-phosphate-guanine
CREB1: cAMP response element-binding protein 1
CRLF1: cytokine receptor-like factor 1
CUT&RUN: cleavage under targets and release using nuclease
CUT&Tag: cleavage under targets and tagmentation
CX3CR1: C-X3-C motif chemokine receptor 1
DNA: deoxyribonucleic acid
DNMT: DNA methyltransferase
DNMTs: DNA methyltransferases
ECM: extracellular matrix
EZH2: enhancer of zeste homolog 2
FRS2: fibroblast growth factor receptor substrate 2
FSP1: ferroptosis suppressor protein 1
GDF6: growth differentiation factor 6
*GPX4*: glutathione peroxidase 4
H3K27ac: histone H3 lysine 27 acetylation
H3K27me3: histone H3 lysine 27 trimethylation
H3K36me2: histone H3 lysine 36 dimethylation
H3K4me1: histone H3 lysine 4 monomethylation
H3K9me3: histone H3 lysine nine trimethylation
HAT: histone acetyltransferase
HATs: histone acetyltransferases
HDAC: histone deacetylase
HDACs: histone deacetylases
Hi-C: high-throughput chromosome conformation capture
HiChIP: high-throughput chromosome conformation capture combined with chromatin immunoprecipitation
HMGB1: high-mobility group box 1
ICAM-1: intercellular adhesion molecule 1
IL-1β: interleukin-1β
IL-6: interleukin-6
IRF1: interferon regulatory factor 1
JAK/STAT: Janus kinase/signal transducer and activator of transcription
KDM3A: lysine demethylase 3A
KDM6B: lysine demethylase 6 B
LDHA: lactate dehydrogenase A
lncRNA: long non-coding RNA
lncRNAs: long non-coding RNAs
m6A: N6-methyladenosine
MAPK: mitogen-activated protein kinase
